# Depression, Anxiety and Associated Factors among Frontline Hospital Healthcare Workers in the Fourth Wave of COVID-19: Empirical Findings from Vietnam

**DOI:** 10.3390/tropicalmed7010003

**Published:** 2021-12-23

**Authors:** Quoc-Hung Doan, Nguyen-Ngoc Tran, Manh-Hung Than, Hoang-Thanh Nguyen, Van-San Bui, Dinh-Hung Nguyen, Hoang-Long Vo, Trong-Thien Do, Ngoc-Thach Pham, Tuan-Khanh Nguyen, Duc-Chinh Cao, Vu-Trung Nguyen, Thin-Mai T. Tran, Ba-Hien Pham, Anh-Long Tran, Van-Thuong Nguyen, Van-Thanh Nguyen, Xuan-Thang Tran, Duc-Truong Lai, Quang-Hieu Vu, Satoko Otsu

**Affiliations:** 1 Department of Surgery, Hanoi Medical University, Hanoi 100000, Vietnam; hung.doanquoc@hmu.edu.vn; 2 Department of Cardiovascular and Thoracic Surgery, Viet Duc University Hospital, Hanoi 100000, Vietnam; 3 Hanoi Medical University Hospital, Hanoi Medical University, Hanoi 100000, Vietnam; 4 Department of Psychiatry, Hanoi Medical University, Hanoi 100000, Vietnam; dotrongthien1794@gmail.com; 5 National Institute of Mental Health, Bach Mai Hospital, Hanoi 100000, Vietnam; 6 Emergency Department, National Hospital Of Tropical Diseases, Hanoi 100000, Vietnam; hungykhoa@gmail.com; 7 Office of Postgraduate Management, Hanoi Medical University, Hanoi 100000, Vietnam; 8 Hanoi Department of Health, Hanoi 100000, Vietnam; ndhung71@gmail.com; 9 Institute for Preventive Medicine and Public Health, Hanoi Medical University, Hanoi 100000, Vietnam; vohoanglonghmu@gmail.com; 10 National Hospital of Tropical Diseases, Hanoi 100000, Vietnam; phamngocthachnhtd@gmail.com (N.-T.P.); ntkhanhdp@gmail.com (T.-K.N.); 11 Ha Dong General Hospital, Hanoi 100000, Vietnam; dr.chinh68hd@gmail.com (D.-C.C.); vutrungy2e@gmail.com (V.-T.N.); 12 Dong Da General Hospital, Hanoi 100000, Vietnam; hoasythanoi@gmail.com (T.-M.T.T.); phambahien.bvdd@gmail.com (B.-H.P.); 13 Duc Giang General Hospital, Hanoi 100000, Vietnam; trananhlong64@gmail.com (A.-L.T.); thuongnhixanhpon@gmail.com (V.-T.N.); 14 North Thang Long Hospital, Hanoi 100000, Vietnam; bsnguyenthanhbvbtl@gmail.com (V.-T.N.); xuanthangbvbtl@gmail.com (X.-T.T.); 15 Disease Control and Health Emergency Program, World Health Organization Vietnam Country Office, Hanoi 100000, Vietnam; laiD@who.int (D.-T.L.); vuh@who.int (Q.-H.V.); otsus@who.int (S.O.)

**Keywords:** COVID-19, psychological impacts, public health, preparedness

## Abstract

(1) Background: This study aims to assess the magnitude of, and factors associated with, depression and anxiety among Vietnamese frontline hospital healthcare workers in the fourth wave of COVID-19; (2) Methods: A hospital based cross-sectional study was carried out within two weeks, October 2020, at a central COVID-19 treatment hospital. Depression and anxiety were measured with PHQ-9 and GAD-7, respectively. Bivariate and multivariate logistic regression analysis were applied to recognize variables related to depression and anxiety, respectively; (3) Results: Among 208 frontline hospital healthcare workers, overall prevalence of depressive symptoms, anxiety symptoms, and both symptoms of depression and anxiety was 38.94%, 25.48% and 24.04%, respectively, in healthcare workers. In a reduced model after using multivariate stepwise logistic regression, age (OR = 0.9, *p* = 0.001), marital status (OR = 7.84, *p* = 0.027), profession (OR = 0.39, *p* = 0.028), having experienced traumatic stress following a work event (OR = 46.24, *p* < 0.001), feeling at very high risk for COVID-19 (OR = 0.02, *p* < 0.04), and affected by workplace conditions (OR = 5.36, *p* < 0.001) were associated with the symptoms of depression. With regard to symptoms of anxiety, single status (OR: 12.18, *p* = 0.002), being medical technician (OR: 68.89, *p* < 0.001), alcohol use (OR: 6.83, *p* = 0.014), using pain relief medications (OR: 25.50, *p* = 0.047), having experienced traumatic stress following a family event (OR: 130.32, *p* = 0.001), having experienced traumatic stress following a work event (OR: 181.55, *p* = 0.002), reporting at very high risk for COVID-19 (OR: 29.64, *p* = 0.011), treating moderate (OR: 6.46, *p* = 0.038) and severe (OR: 18.96, *p* = 0.004) COVID-19 patients, and being significantly affected by the community (OR: 6.33, *p* = 0.003) were increased risk factors for the symptoms of anxiety. Meanwhile, those living with 4–5 people (OR: 0.15, *p* = 0.011), specializing in infectious disease (OR: 0.13, *p* = 0.044)/resuscitation and emergency medicine (OR: 0.04, *p* = 0.046), and having knowledge preparation before participating in COVID-19 (OR: 0.008, *p* = 0.014) were less associated with the symptoms of anxiety; (4) Conclusions: There was a relatively high prevalence among Vietnamese hospital healthcare workers exhibiting symptoms of depression and anxiety during the ongoing pandemic. Greater attention to training in psychological skills should be suggested for those belonging to a younger age group, being single/widowed/divorced, treating moderate and severe COVID-19 patients, feeling at very high risk for COVID-19, being significantly affected a lot the community or workplace conditions, or experiencing traumatic stress following a family/work event in the past week.

## 1. Introduction

With the rapid spread of SARS-CoV-2, health care resource responsiveness challenges are posed to health systems globally [1]. Especially when high rates of COVID-19 infection are reported among healthcare workers [2,3,4], with the increase in SARS-CoV-2-related mortalities in the general population, anxiety and depression tended to be common psychological problems in healthcare workers [5]. Medical staff not only have to work overtime compared to their working time as before the COVID-19 epidemic, but also have a high risk of virus infection during the care and treatment of COVID-19 patients [6,7]. Besides, prolonged stress also contributes to an increased likelihood of depression or other mental disorder, leading to an increased risk of infection and disease severity [8,9]. In a recent systematic review of updated prevalence estimates for depression and anxiety from 65 studies, Yufei Li showed a high prevalence of moderate depression and anxiety among health care workers across 21 countries during the COVID-19 pandemic [10], which can negatively impact on the quality of COVID-19 patient care [11]. In Southeast Asia alone, recent evidence has revealed that there seems to be an increasing trend for anxiety and depression over time among healthcare workers compared to the first wave of COVID-19 [12,13,14,15,16].

Frontline healthcare workers are at high-risk of acquiring SARS-CoV-2 infection during medical procedures due to their close contact with highly infectious patients, particularly those who are in COVID-19 patient-treatment isolation zones [17,18,19]. There is currently no clarity regarding the estimates of the prevalence of depression and anxiety among medical staff working in isolation treatment facilities for COVID-19 patients, who known as frontline hospital healthcare workers, limiting the possibility of informing action in policy and practice to perform targeted psychological interventions for health care workers during this time of crisis. The impact of the fourth wave of COVID-19 in Vietnam was extremely severe with the emergence of the dangerous Delta variant of the SARS-CoV-2 virus, which reversed Vietnam’s epidemic prevention and control achievements in previous COVID-19 waves. The recent wave of the COVID-19 pandemic in Vietnam significantly exceeded the aforementioned previous three pandemic phases in many aspects. There are few studies from different settings of the psychological burden of the Vietnamese healthcare workforce during early national waves of the COVID-19 pandemic, indicating moderately severe depression symptoms, anxiety symptoms, stress and insomnia in healthcare professionals [20,21,22], suggesting initial negative psychological responses among the healthcare workforce; nevertheless, there was no understanding of the psychological issues surrounding the medical staff involved in direct treatment of COVID-19 patients. Moreover, continuous monitoring of the psychological consequences for this high-risk population should become routine as part of targeted interventions during times of crisis because unforeseen changes and the impact of psychological problems are different in each particular context. In the face of long work shifts (that reach 16 h per day on average), the risk of getting infected by a highly infectious disease and the lack of sufficient biological protection measures, mental suffering among health professionals suddenly became evident. Due to this situation in the fourth national COVID-19 wave, we conducted a cross-sectional study at a central COVID-19 treatment hospital in the Northern region of Vietnam to evaluate the prevalence of the symptoms of anxiety and depression of frontline hospital healthcare workers who are working in COVID-19 treatment isolation zones. We further explore the risk factors and protective factors for symptoms of anxiety and depression.

## 2. Methods

### 2.1. Study Design and Participants

We carried out a hospital-based cross-sectional study of the healthcare workforce who worked at the National Hospital of Tropical Diseases (base 2, Hanoi, Vietnam) between 1 October 2021 and 20 October 2021. To foster the engagement of the healthcare workforce, a convenience sampling method was employed for this study, appropriate due to its rapid nature and low-cost given our resource-scarce research setting. Eligibility criteria specified that participants in the study should be: (1) aged from 18 and over; (2) hospital healthcare workers who had obtained a contract to work full-time or part-time at the hospitals, including medical doctors, nurses, midwives, and technicians; (3) involved in the direct treatment of COVID-19 patients and (4) agreed to participate in the survey by providing an informed consent.

### 2.2. Outcome Measurements

The study questionnaire was developed by a group of psychiatrists from the National Institute of Mental Health (Hanoi, Vietnam) and public health experts from the Hanoi Medical University (Hanoi, Vietnam) to collect potential data on profession-related and socio-demographic characteristics, psychological trauma in the past week, COVID-19 control and prevention-related characteristics and psychological status of these hospital healthcare workers. Participants’s psychological problems were assessed with the use of the Vietnamese versions of the 9-item Patient Health Questionnaire (PHQ-9), and the 7-item Generalized Anxiety Disorder-7 (GAD-7) scale. PHQ-9 and GAD-7 are common instruments and easily used to measure and screen the overall presence and level of depression and anxiety. 

Then, the developed questionnaire was piloted on a sample of 20 respondents to test its validity. The primary data was collected via sending the invitation directly to the participants, utilizing structured self-completed questionnaires in the Vietnamese version. No material incentives were suggested to the respondents for their engagement in the survey to avoid them from answering more than once. Final analysis did not include the data from the pilot survey.

- PHQ-9

Depression and degree of depression severity were measured using the PHQ-9, a shorter version of the complete PHQ, where individuals were asked how often they were bothered by various problems within the past two weeks. The nine items of PHQ-9 were ‘Little interest or pleasure in doing things’, ‘Feeling down, depressed, or hopeless’, ‘Trouble falling or staying asleep, or sleeping too much’, ‘Feeling tired or having little energy’, ‘Poor appetite or overeating’, ‘Feeling bad about yourself—or that you are a failure or have let yourself or your family down’, ‘Trouble concentrating on things, such as reading the newspaper or watching television’, ‘Moving or speaking so slowly that other people could have noticed, or so fidgety or restless that you have been moving a lot more than usual’, ‘Thoughts that you would be better off dead, or thoughts of hurting yourself in some way’. Each item was selected, with four-point-scale based answers ranging from 0 (not at all) to 3 (nearly every day). The total score of the PHQ-9 scale after self-reported response ranges from 0 to 27, and more severe depression symptoms are shown by a higher score. Symptom severity was based on the total score and was categorized as follows: absence of depression (0–4), mild depression (5–9), moderate depression (10–14), and severe depression (15–27). In various medical settings, the validated depression scale was reported with good reliability (Cronbach’s α = 0.86–0.89).

- GAD-7

Anxiety was measured using the GAD-7. The GAD-7 scale is a self-reported anxiety questionnaire including seven items ‘Feeling nervous, anxious or on edge’, ‘Not being able to stop or control worrying’, ‘Worrying too much about different things’, ‘Trouble relaxing’, ‘Being so restless that it is hard to sit still’, ‘Becoming easily annoyed or irritable’, ‘Feeling afraid as if something awful might happen’. All the items were rated on a four-point scale scoring from 0 (not at all) to 3 (nearly every day). The total score ranges from 0 to 21, and symptom severity was interpreted as follows: absence of anxiety (0–4), mild anxiety (5–9), moderate anxiety (10–14), and severe anxiety (15–21). Though initially designed to identify generalized anxiety disorder (GAD), the GAD-7 has also been considered as a good screening tool for other common anxiety disorders. The GAD-7 was proved valid with high reliability (Cronbach’s α = 0.89).

### 2.3. Dependent and Independent Variables

We considered clinically significant depression and clinically significant anxiety as binary dependent variables. Clinically significant depression was defined as that in which an individual had a PHQ-9 score of ≥5. Clinically significant anxiety was defined as that in which an individual had a GAD-7 score of ≥5.

Description of independent variables is presented in Table 1. The list of independent variables was based on psychiatric judgment and a literature review.

Profession-related and socio-demographic variables included age, gender, marital status, number of people lived with, family household with own children under 18 years, family household with older person above 60 years, education, profession, medical specialty, alcohol, smoking, comorbidities, and using pain relief medications.

Psychological trauma-related characteristics: hospital health workers were asked whether they had experienced traumatic stress in the past week, including due to family, work, academic, social, disease and economic events.

COVID-19 control and prevention-related characteristics included the severity of the COVID-19 patients who treated, duration of participation in COVID-19 control, knowledge preparation before participation, full equipment in current workplace, being affected by workplace conditions, being affected by the community, feelings regarding COVID-19 infection risk, and having a relative/friend/colleague positive for COVID-19.

### 2.4. Data Analysis

The data obtained was entered in EpiData 3.1, and responses were coded appropriately before being exported to Stata^®^ 15 (StataCorp LLC, College Station, TX, USA) for analysis. Descriptive statistical analysis was first used to characterize the samples of hospital healthcare workers by profession-related and socio-demographic variables, psychological trauma-related characteristics and COVID-19 control and prevention work-related characteristics. Frequencies and proportions for each categorical variable were calculated and described, while quantitative variables were expressed as mean, standard deviation (SD) and interquartile range (IQR). Bivariate logistic regression analyses were used to examine the associations between all variables of interest and the two outcomes. Both univariate and multivariate logistic regression models were used to identify the associations between profession-related and socio-demographic variables, psychological trauma-related characteristics and COVID-19 control and prevention-related characteristics and the two outcome variables of clinically significant depression and anxiety, respectively. Finally, a total of valid variables that were considered as independent variables (work-related and socio-demographic variables, psychological trauma-related characteristics and COVID-19 control and prevention-related characteristics) were put into a full model for multivariate logistic regression analysis. A stepwise backward selection strategy with *p* values < 0.2 was applied, and then two reduced models with multivariable logistic regression were established for clinically significant depression and anxiety, respectively. A *p*-value < 0.05 was considered to be statistically significant.

## 3. Results

In total, the responses of 208 hospital healthcare workers were included in the final analysis between 1 October 2021 and 20 October 2021.

Table 2 summarizes the profession-related and socio-demographic characteristics of the hospital healthcare workers. There were 79 (37.98%) male and 129 (62.02%) female respondents. The majority of the participants were married (75.00%), were a medical doctor or nurse/midwife (85.09%), and had an educational level of university and post-graduate (49.52%). Respectively, 67.31% and 27.40% reported a family household with own children under 18 years, and with older relative above 60 years. The distribution of medical speciality groups included 28.37% infectious disease, 9.13% resuscitation and emergency medicine, 11.54% surgery, 7.69% internal medicine, and 5.29% anesthesiology. Most were living with 1–5 people (75.48%). Self-reported alcohol and smoking were documented in 50.00% and 13.94%, respectively. The prevalence of non-psychiatric comorbidities was 48.08% in participants, and 2.88% had been using pain relief medications.

Regarding COVID-19 control and prevention work-related characteristics, a total of 61 of 208 healthcare workers (29.33%) participated in the treatment of severe COVID-19 patients, and 74 (35.58%) were involved in the treatment of moderate patients. Most had participated in controlling COVID-19 for over 1 month (85.10%). The majority of healthcare workers had obtained relevant knowledge before participating in COVID-19 care (94.23%), and reported with full equipment in the current workplace conditions (93.75%). Of healthcare workers, 38.46% were affected by workplace conditions, and 37.98% were influenced significantly by the community. A feeling of high and very high risk from COVID-19 was common in participants (62.98%) and 52.88% of healthcare workers had a relative/friend/colleague with positive COVID-19 (Table 2). As was shown in Table 2, the most common traumatic stress among medical staff followed an economic event (17.79%) or a family event (10.58%).

### 3.1. Mental Health Status

Table 3 depicts the percentage of respondents by level of depression and anxiety during the fourth wave of COVID-19 in Vietnam. Of the 208 participants, 38.94% of them reported symptoms of depression, 25.48% reported symptoms of anxiety, and 24.04% reported both symptoms of depression and anxiety. Results found that 3.85% of the hospital healthcare workers reported severe depression, 7.69% reported moderately severe depression and more than one-fourth (27.40%) reported mildly severe depression. Of the participants, 24.52% had mild and severe anxiety symptoms, and only 2 (0.96%) respondents had severe depression symptoms. 24.04% had undergone both depression and anxiety.

**Figure 1:**
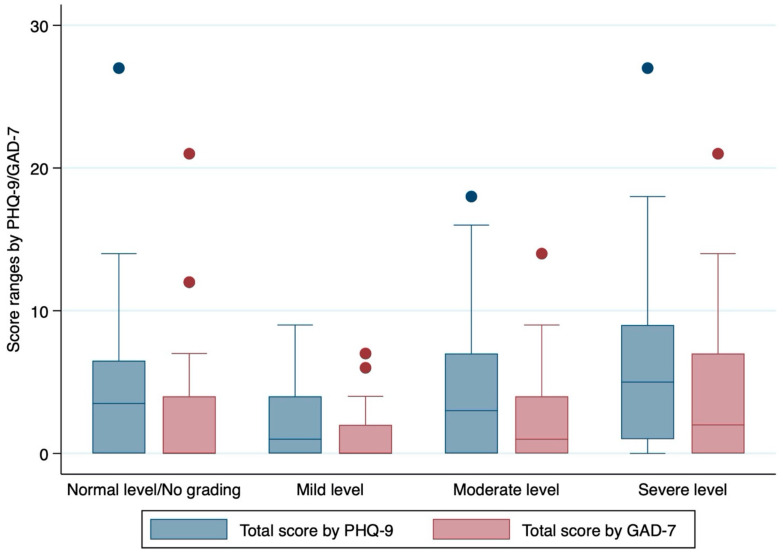
Measurement of total score by PHQ-9 and total score by GAD-7 according to the severity of COVID-19 patient who were treated by hospital health workers.

Especially, statistically significant difference in total score by PHQ-9 (*p* = 0.0202) and total score by GAD-7 (*p* = 0.0011) were observed amongst the severity levels of COVID-19 patients. Both depression score by PHQ-9 and anxiety score by GAD-7 were highest in the severe group (Figure 1).

### 3.2. Association with Symptoms of Depression

Table 4 indicates analysis result of factors associated with depression using univariable and multivariable logistic regression. Statistically significant variables which were associated with depression in both logistic regressions included medical staff’s age (OR univariable: 0.93, 95%CI 0.88–0.97; OR multivariable: 0.88, 95%CI 0.81–0.97), having experienced traumatic stress following a work event in the past week (OR univariable: 11.53, 95%CI 4.20–31.62; OR multivariable: 298.08, 95%CI 14.99–5926.01), having experienced traumatic stress following a disease event in the past week (OR univariable: 10.08, 95%CI 1.19–85.35; OR multivariable: 136.42, 95%CI 1.57–11,792.85), duration of participation in COVID-19 control within 1–3 months (OR univariable: 0.29, 95%CI 0.12–0.72; OR multivariable: 0.21, 95%CI 0.05–0.86), and being affected by workplace conditions (OR univariable: 3.93, 95%CI 2.17–7.12; OR multivariable: 4.50, 95%CI 1.63–12.39).

### 3.3. Association of the Symptoms of Anxiety

In the reduced model after using multivariate stepwise logistic regression (Table 5), we found age, marial status, profession, having experienced traumatic stress following a work event, feeling at very high risk for COVID-19, and being affected by workplace conditions were associated with clinically significant depression in hospital healthcare workers. Older age was associated with a lower risk of depression (OR = 0.9, 95%CI: 0.85–0.96, *p* = 0.001). The prevalence of depression symptoms in the widowed/divorced group was higher than in the married group (OR = 7.84, 95%CI: 1.26–48.60, *p* = 0.027). Compared to respondents who were medical doctors, those being a medical technician was associated with lower risks of depression (OR = 0.39, 95%CI: 0.17–0.90, *p* = 0.028). Those with traumatic stress following a work event in the past week had higher risk of depression than those without traumatic stress following a work event (OR = 46.24, 95%CI: 9.12–234.28, *p* < 0.001). Those who felt at very high risk for COVID-19 had lower risk of depression compared to those reporting no infected risk (OR = 0.02, 95%CI: 0.0005–0.83, *p* < 0.04). Individuals affected by workplace conditions had an elevated risk for depression (OR = 5.36, 95%CI: 2.41–11.92, *p* < 0.001).

In both univariate and multivariable analysis (Table 6), single status (OR univariable: 2.44, 95%CI 1.18–5.03; OR multivariable: 7.28, 95%CI 1.03–51.24), having experienced traumatic stress following a family event (OR univariable: 10.73, 95%CI 3.93–29.33; OR multivariable: 153.97, 95%CI 5.43–4362.13), having experienced traumatic stress following a work event (OR univariable: 17.49, 95%CI 6.89–44.40; OR multivariable: 265.42, 95%CI 8.39–8389.72), and being significantly affected by the community (OR univariable: 4.90, 95%CI 2.51–9.55; OR multivariable: 6.13, 95%CI 1.40–26.84) were found to be associated with the symptoms of anxiety.

The results from the multivariate stepwise logistic regression are presented in Table 6. Single status (OR: 12.18, 95%CI 2.48–59.85, *p* = 0.002), being a medical technician (OR: 68.89, 95%CI 7.33–646.98, *p* < 0.001), alcohol intake (OR: 6.83, 95%CI 1.48–31.58, *p* = 0.014), using pain relief medication (OR: 25.50, 95%CI 1.04–620.52, *p* = 0.047), having experienced traumatic stress following a family event (OR: 130.32, 95%CI 7.06–2404.04, *p* = 0.001), having experienced traumatic stress following a work event (OR: 181.55, 95%CI 8.80–3745.22, *p* = 0.002), reporting at very high risk for COVID-19 (OR: 29.64, 95%CI 2.20–398.16, *p* = 0.011), treating moderate (OR: 6.46, *p* = 0.038) and severe (OR: 18.96, *p* = 0.004) COVID-19 patients, and being significantly affected by the community (OR: 6.33, 95%CI 1.89–21.19, *p* = 0.003) were increasing risk factors for the symptoms of anxiety in hospital healthcare workers. Meanwhile, those living with 4–5 people (OR: 0.15, 95%CI 0.03–0.65, *p* = 0.011), specializing in infectious diseases (OR: 0.13, 95%CI 0.01–0.94, *p* = 0.044)/resuscitation and emergency medicine (OR: 0.04, 95%CI 0.002–0.94, *p* = 0.046), and obtaining relevant knowledge before participating in COVID-19 treatment (OR: 0.008, 95%CI 0.0002–0.37, *p* = 0.014) were less associated with the symptoms of anxiety.

## 4. Discussion

Despite the research regarding the various impact of COVID-19 on healthcare worker wellness, little is currently known about psychological impacts of the COVID-19 pandemic on the medical staff involved in direct treatment of COVID-19 patients in isolation treatment zones which can be aggregated to assess prevalence accurately and to provide a complete understanding of the effectiveness of psychological interventional strategies. The present study, promptly carried out during the ongoing COVID-19 pandemic in Vietnam, investigated the prevalence of and risk/protective factors associated with depression and anxiety symptoms among hospital healthcare workers who are working in COVID-19 treatment facilities based on a health facility convenient-sample survey. Approximately two-fifth (38.94%) and one-fourth (25.48%) of healthcare workers exhibited symptoms of depression and anxiety, respectively, while nearly one-fourth (24.04%) of them documented both symptoms of depression and anxiety. In fact, the rates of depression and anxiety in this study were not higher than those reported previously. This can be understood due to the long-term adaptive response to the fight against the COVID-19 epidemic of the Vietnamese health system in general, as well as frontline medical staff in particular. Especially, the healthcare workforce who have been working at the National Hospital of Tropical Diseases were involved in the treatment of COVID-19 from the first cases in the first wave of COVID-19 pandemic, and so by the current fourth COVID-19 wave in Vietnam had extensive experience in managing COVID-19 patients in isolation treatment areas. Several psychologically vulnerable populations were also identified, such as individuals with single/widowed/divorced status, those who had experienced traumatic stress following a work event in the past week, those who were treating moderate and severe COVID-19 patients, and those who were significantly affected by the community. These findings contributed to the building of clear strategies to support and appropriately manage hospital healthcare workers involved in the treatment of COVID-19 patients, essential to ensure effective staff management and to engender trust in isolation treatment zones.

The result suggests that feeling at very high risk for COVID-19 is a critical factor in understanding the increased prevalence of depression and anxiety among participants who were working in isolation COVID-19 treatment zones. This finding is in accord with previous evidence reporting that doctors and nurses working in high-risk departments had higher risk of at least one mental health problem [23]. With the rapid increase in the number of hospitalized COVID-19 patients, medical staffs have to face enormous workload and high-risk of infection [24], which easily leads to work trauma for the COVID-19 treatment staff team. One of our findings was consistent with this statement, as higher levels of anxiety/depression were also documented among those who reported with traumatic stress following a work event in the past week.

Our findings indicate thatadvanced age was a protective factor for depression symptoms, but this age variable is not statistically significant for anxiety related models in all present analyses. In the Egyptian population, age was reported to show a significantly negative correlation with depression during the COVID 19 outbreak [25]. A systematic review of Jiaqi Xiong also showed that those from the younger age group (≤40 years) presented with more depressive symptoms [26]. Compared healthcare workers only, our finding was consistent with previous reports [27]. In addition, with respect to marital status, this was identified as associated with the prevalence of depression and anxiety in hospital healthcare workers. Herein, the prevalence of depression/anxiety symptoms in those being widowed/divorced was higher than in those who were married. There was an association of marital status with depressive symptoms in healthcare workers in Di Tella’s study [28], while one other study reported that married people had higher levels of anxiety when compared to those unmarried [29].

Usually, most medical staff working in hospitals had not receivedd mental health training, and consequently daily working hours were positively associated with all psychological disorders in frontline healthcare workers, such as depression and anxiety, [27,30], especially worrisome in hospital health professionals who were involved in treating moderate and severe COVID-19 patients. We found that treating moderate and severe COVID-19 patients was a predictor for clinically significant anxiety. The reason may be that hospital medical staffs facing severely infected patients must regularly monitor, as well as worry about the worsening of, these severe cases, which is clearly different to healthcare workers who managed mild cases with no symptoms.

Several implications can be inferred from these results. It seems that the symptoms of depression and anxiety during the COVID-19 pandemic for frontline healthcare workers are mainly caused as a response to the life-threatening situation and being placed under significant pressure. At the family and social level, a psychological counseling hotline should be widely opened with the support of family members, psychological doctors, social workers, and volunteers.

The strengths of the current study are determined by several issues. To date, no updated report of the prevalence of anxiety and depression during the fourth wave of COVID-19 has been published in Vietnam. Despite caveats, the present study provides insights into the work-related and socio-demographic factors, psychological trauma-related factors and COVID-19 control and prevention work-related factors and the symptoms of depression, and is the first study in Vietnam indicating relative prevalence of clinically significant depression and anxiety in a particular healthcare population.

The study limitations should, however, also be noted before interpretation. First, the PHQ-9 and GAD-7 have been, in fact, less commonly applied to ascertain population or community prevalence of depression symptoms or generalised anxiety symptoms. The present study did not establish the sensitivity, specificity and positive and negative predictive values of cut-off scores using the PHQ-9 and GAD-7 with health workers. Second, self-reported alcohol and tobacco consumption, in addition, comes with an inherent limitation due to no measurement with specific instruments for the two variables. Third, owing to the COVID-19 urgency and the time limit, the frontline medical staff involved in direct treatment of COVID-19 patients in isolation treatment zones might have expressed less depression and anxiety than the actual condition, due to social desirability factors. Fourth, the survey’s timing may limit generalization to all hospital healthcare workers who were working during fourth COVID-19 epidemic period and in other parts of Vietnam where the pandemic situation was more severe such as Ho Chi Minh City and western provinces of Vietnam. Finally, our sample size is not large enough to represent COVID-19 treatment facilities with the cross-sectional design used, which may also have limited statistical power to detect differential associations with the severity of depressive and anxiety symptoms. Given the time-sensitivity of the COVID-19 outbreak and limited resources available, the study was not distributed to wider, similar populations in other COVID-19 hotspots.

## 5. Conclusions

This study provides the first empirical evidence of the relative prevalence among Vietnamese hospital healthcare workers of symptoms of depression and anxiety during the ongoing pandemic. Training in psychological skills for individuals belonging to younger age groups, being single/widowed/divorced, treating moderate and severe COVID-19 patients, feeling very high COVID-19 infection risk, being significantly affected a lot by community/workplace conditions, and experiencing traumatic stress following a family/work event in the past week should be studied further to ensure the continuous involvement of the hospital healthcare workforce in COVID-19 patient management and treatment in isolation health facilities.

## Figures and Tables

**Table 1:** Description of independent variables.

Variable Name	Variable Label	Value Label	Types of Variable
Profession-Related and Socio-Demographic Variables			
A1	Age	Years	Quantitative variable (Discrete)
A2	Gender	1 = male; 2 = female	Qualitative variable (Binary)
A3	Marital status	1 = married; 2 = single; 3 = widowed/divorced	Qualitative variable (Nominal)
A4	Number of people living with	People	Quantitative variable (Discrete)
A5	Family household with own children under 18 years	1 = no; 2 = yes	Qualitative variable (Binary)
A6	Family household with own older person above 60 years	1 = no; 2 = yes	Qualitative variable (Binary)
A7	Education	1 = lower secondary/upper secondary; 2 = college; 3 = university; 4 = postgraduation	Qualitative variable (Nominal)
A8	Profession	1 = medical doctor; 2 = nurse and midwife; 3 = others	Qualitative variable (Nominal)
A9	Medical specialty	1 = internal medicine; 2 = surgery; 3 = infectious disease; 4 = resuscitation and emergency medicine; 5 = anesthesiology; 6 = others	Qualitative variable (Nominal)
A10	Alcohol	1 = no; 2 = yes	Qualitative variable (Binary)
A11	Smoking	1 = no; 2 = yes	Qualitative variable (Binary)
A12	Comorbidities	1 = no; 2 = yes	Qualitative variable (Binary)
A13	Using pain relief medications	1 = no; 2 = yes	Qualitative variable (Binary)
**Psychological trauma-related characteristics**			
B1	Having experienced traumatic stress following a family event	1 = no; 2 = yes	Qualitative variable (Binary)
B2	Having experienced traumatic stress following a work event	1 = no; 2 = yes	Qualitative variable (Binary)
B3	Having experienced traumatic stress following an academic event	1 = no; 2 = yes	Qualitative variable (Binary)
B4	Having experienced traumatic stress following a social event	1 = no; 2 = yes	Qualitative variable (Binary)
B5	Having experienced traumatic stress following a disease event	1 = no; 2 = yes	Qualitative variable (Binary)
B6	Having experienced traumatic stress following an economic event	1 = no; 2 = yes	Qualitative variable (Binary)
**COVID-19 control and prevention-related characteristics**			
C1	Severity of COVID-19 patients who were treated	1 = normal level; 2 = mild level; 3 = moderate level; 4 = severe level	Qualitative variable (Ordinal)
C2	Duration participating in COVID-19 control	Months	Quantitative variable (Discrete)
C3	Knowledge preparation before participating in COVID-19	1 = no; 2 = yes	Qualitative variable (Binary)
C4	Full equipment in current workplace conditions	1 = no; 2 = yes	Qualitative variable (Binary)
C5	Affected by workplace conditions	1 = no; 2 = yes	Qualitative variable (Binary)
C6	Affected a lot by the community	1 = no; 2 = yes	Qualitative variable (Binary)
C7	Feeling with COVID-19 infection risk	1 = no risk; 2 = low risk; 3 = average risk; 4 = high risk; 5 = very high risk; 6 = infected	Qualitative variable (Ordinal)
C8	Having a relative/friend/colleague with positive COVID-19	1 = no; 2 = yes	Qualitative variable (Binary)

**Table 2:** Profession-related and socio-demographic characteristics of hospital health workers.

Profession-Related and Socio-Demographic Characteristics		*N* = 208	Percentage (%)
Age—Mean; SD (IQR)		33.20; 6.77 (22–60)	
Gender	Male	79	37.98
	Female	129	62.02
Marital status	Married	156	75.00
	Single	42	20.19
	Widowed/Divorced	10	4.81
Number of people living with (people)	1–3 people	43	20.67
	4–5 people	114	54.81
	>5 people	51	24.52
Family household with own children under 18 years	No	68	32.69
	Yes	140	67.31
Family household with own older person above 60 years	No	151	72.60
	Yes	57	27.40
Education	Lower secondary/upper secondary	10	4.81
	College	95	45.67
	University	64	30.77
	Postgraduation	39	18.75
Profession	Medical doctor	57	27.40
	Nurse and midwife	120	57.69
	Medical technician	31	14.90
Medical specialty	Internal medicine	16	7.69
	Surgery	24	11.54
	Infectious disease	59	28.37
	Resuscitation and emergency medicine	19	9.13
	Anesthesiology	11	5.29
	Others	79	37.98
Alcohol	No	104	50.00
	Yes	104	50.00
Smoking	No	179	86.06
	Yes	29	13.94
Comorbidities	No	108	51.92
	Yes	100	48.08
Using pain relief medications	No	202	97.12
	Yes	6	2.88
**COVID-19 control and prevention-related characteristics**			
Severity of COVID-19 patient	Normal level	32	15.38
	Mild level	41	19.71
	Moderate level	74	35.58
	Severe level	61	29.33
Duration participating in COVID-19 control (months)	<1 month	31	14.90
	1–3 month(s)	62	29.81
	>3 months	115	55.29
Knowledge preparation before participating in COVID-19	No	12	5.77
	Yes	196	94.23
Full equipment in current workplace conditions	No	13	6.25
	Yes	195	93.75
Affected by workplace conditions	No	128	61.54
	Yes	80	38.46
Affected a lot by the community	No	129	62.02
	Yes	79	37.98
Feeling with COVID-19 infection risk	No risk	22	10.58
	Low risk	55	26.44
	Average risk	54	25.96
	High risk	49	23.56
	Very high risk	26	12.50
	Infected	2	0.96
Having a relative/friend/colleague with positive COVID-19	No	98	47.12
	Yes	110	52.88
**Psychological trauma-related characteristics in the past one week**			
Having experienced traumatic stress following a family event	No	186	89.42
	Yes	22	10.58
Having experienced traumatic stress following a work event	No	177	85.10
	Yes	31	14.90
Having experienced traumatic stress following an academic event	No	198	95.19
	Yes	10	4.81
Having experienced traumatic stress following a social event	No	193	92.79
	Yes	15	7.21
Having experienced traumatic stress following a disease event	No	201	96.63
	Yes	7	3.37
Having experienced traumatic stress following an economic event	No	171	82.21
	Yes	37	17.79

SD: standard deviation; IQR: interquartile range.

**Table 3:** Prevalence of depression and anxiety among hospital health workers.

		*N* = 208
Depression by PHQ-9—Frequency (%)	Absence of depression	127 (61.06)
	Mild depression	57 (27.40)
	Moderate depression	16 (7.69)
	Severe depression	8 (3.85)
Total score by PHQ-9—Mean, SD (IQR)		4.31, 4.83 (0–27)
Anxiety by GAD-7	Absence of anxiety	155 (74.52)
	Mild anxiety	44 (21.15)
	Moderate anxiety	7 (3.37)
	Severe anxiety	2 (0.96)
*Total score by GAD-7*—Mean, SD (IQR)		2.67, 3.76 (0–21)
Both depression and anxiety—Frequency (%)		50 (24.04)

SD: standard deviation; IQR: interquartile range.

**Table 4:** Analysis of factors associated with the symptoms of depression: univariable and multivariable logistic regression.

		Clinically Significant Depression		Univariable				Multivariable			
		No (*N* = 127)	Yes (*N* = 81)	OR	*p*-Value	Confidence Interval 95%		OR	*p*-Value	Confidence Interval 95%	
						Lower	Upper			Lower	Upper
**Work-Related and Socio-Demographic Variables**											
Age (Mean; SD)		34.35 (6.80)	31.41 (6.39)	0.93	0.003 **	0.88	0.97	0.88	0.010 *	0.81	0.97
Gender	Male (ref)	44	35								
	Female	83	46	0.69	0.215	0.39	1.23	1.83	0.310	0.56	5.90
Marital status	Married (ref)	100	56								
	Single	23	19	1.47	0.270	0.73	2.94	0.53	0.316	0.15	1.83
	Widowed/Divorced	4	6	2.67	0.139	0.72	9.89	13.34	0.032 *	1.25	142.10
Number of people living with (people)	1–3 people (ref)	22	21								
	4–5 people	73	41	0.58	0.143	0.28	1.19	0.87	0.834	0.24	3.06
	>5 people	32	19	0.62	0.259	0.27	1.41	1.25	0.765	0.27	5.68
Family household with own children under 18 years	No (ref)	37	31								
	Yes	90	50	0.66	0.172	0.36	1.19	0.87	0.805	0.29	2.57
Family household with own older person above 60 years	No (ref)	89	62								
	Yes	38	19	0.71	0.309	0.37	1.35	0.56	0.316	0.18	1.72
Education	Lower secondary/upper secondary (ref)	7	3								
	College	54	41	1.77	0.427	0.43	7.27	1.05	0.965	0.07	14.13
	University	42	22	1.22	0.786	0.28	5.19	0.62	0.719	0.04	8.05
	Postgraduation	24	15	1.45	0.622	0.32	6.52	0.60	0.729	0.03	10.14
Profession	Medical doctor (ref)	30	27								
	Nurse and midwife	77	43	0.62	0.144	0.32	1.17	0.244	0.137	0.03	1.56
	Medical technician	20	11	0.61	0.284	0.24	1.50	0.82	0.860	0.10	6.78
Medical specialty	Internal medicine (ref)	12	4								
	Surgery	9	15	4.99	0.024	1.23	20.30	3.10	0.280	0.39	24.20
	Infectious disease	41	18	1.31	0.668	0.37	4.64	0.60	0.618	0.08	4.43
	Resuscitation and emergency medicine	9	10	3.33	0.103	0.78	14.15	1.35	0.806	0.12	14.88
	Anesthesiology	5	6	3.59	0.126	0.69	18.55	0.86	0.917	0.06	11.88
	Others	51	28	1.64	0.423	0.48	5.58	1.31	0.779	0.19	9.14
Alcohol	No (ref)	67	37								
	Yes	60	44	1.32	0.320	0.75	2.32	1.75	0.305	0.59	5.15
Smoking	No (ref)	110	69								
	Yes	17	12	1.12	0.772	0.50	2.49	1.25	0.742	0.31	4.99
Comorbidities	No (ref)	70	38								
	Yes	57	43	1.38	0.249	0.79	2.43	1.35	0.578	0.46	3.93
Using pain relief medications	No (ref)	126	76								
	Yes	1	5	8.28	0.056	0.95	72.29	9.56	0.176	0.36	251.95
**Psychological trauma-related variables in the past one week**											
Having experienced traumatic stress following a family event	No (ref)	120	66								
	Yes	7	15	3.89	0.005 **	1.51	10.03	0.35	0.423	0.02	4.53
Having experienced traumatic stress following a work event	No (ref)	122	55								
	Yes	5	26	11.53	0.000 ***	4.20	31.62	298.08	0.000 ***	14.99	5926.01
Having experienced traumatic stress following an academic event	No (ref)	123	75								
	Yes	4	6	2.46	0.174	0.67	9.00	0.03	0.096	0.0006	1.82
Having experienced traumatic stress following a social event	No (ref)	122	71								
	Yes	5	10	3.43	0.030 *	1.12	10.45	0.220	0.278	0.01	3.38
Having experienced traumatic stress following a disease event	No (ref)	126	75								
	Yes	1	6	10.08	0.034 *	1.19	85.35	136.42	0.031 *	1.57	11,792.85
Having experienced traumatic stress following an economic event	No (ref)	115	56								
	Yes	12	25	4.28	0.000 ***	2.00	9.13	1.42	0.677	0.26	7.66
**COVID-19 control and prevention-related variables**											
Severity of COVID-19 patient	Normal level (ref)	21	11								
	Mild level	31	10	0.61	0.352	0.22	1.70	0.77	0.757	0.14	3.99
	Moderate level	45	29	1.23	0.639	0.51	2.92	1.30	0.713	0.31	5.37
	Severe level	30	31	1.97	0.133	0.81	4.78	2.43	0.259	0.51	11.46
Duration participating in COVID-19 control (months)	<1 month (ref)	13	18								
	1–3 month(s)	44	18	0.29	0.008 **	0.12	0.72	0.21	0.030 *	0.05	0.86
	>3 months	70	45	0.46	0.062	0.20	1.03	0.396	0.200	0.09	1.63
Knowledge preparation before participating in COVID-19	No (ref)	4	8								
	Yes	123	73	0.29	0.054	0.08	1.01	0.12	0.091	0.01	1.38
Full equipment in current workplace conditions	No (ref)	7	6								
	Yes	120	75	0.72	0.583	0.23	2.25	1.38	0.746	0.18	10.16
Affected by workplace conditions	No (ref)	94	34								
	Yes	33	47	3.93	0.000 ***	2.17	7.12	4.50	0.004 **	1.63	12.39
Affected a lot by the community	No (ref)	93	36								
	Yes	34	45	3.41	0.000 ***	1.89	6.15	1.47	0.416	0.57	3.79
Feeling with COVID-19 infection risk	No risk (ref)	15	7								
	Low risk	35	20	1.22	0.706	0.42	3.50	3.99	0.166	0.56	28.29
	Average risk	31	23	1.58	0.385	0.55	4.52	3.228	0.221	0.49	21.05
	High risk	32	17	1.13	0.813	0.38	3.32	2.77	0.311	0.38	20.00
	Very high risk	13	13	2.14	0.206	0.65	6.98	3.05	0.352	0.28	32.28
	Infected	1	1	2.14	0.608	0.11	39.46	0.06	0.264	0.0005	7.843
Having a relative/friend/colleague with positive COVID-19	No (ref)	64	34								
	Yes	63	47	1.40	0.236	0.80	2.46	0.53	0.216	0.19	1.44
Pseudo R2								0.4054			

OR: odd ratio; *, **, ***: significant at 0.05, 0.01 and 0.001.

**Table 5:** Analysis of factors associated with the symptoms of depression and anxiety, respectively: multivariate stepwise logistic regression.

		Clinically Significant Depression				Clinically Significant Anxiety			
		OR	*p*-Value	Confidence Interval 95%		OR	*p*-Value	Confidence Interval 95%	
				Lower	Upper			Lower	Upper
**Profession-Related and Socio-Demographic Variables**									
Age		0.90	0.001 **	0.85	0.96				
Marial status	Married (ref)								
	Single					12.18	0.002 **	2.48	59.85
	Widowed/Divorced	7.84	0.027 *	1.26	48.60	15.03	0.089	0.66	341.68
Number of people living with (people)	1–3 people (ref)								
	4–5 people					0.15	0.011 *	0.03	0.65
	>5 people								
Family household with own older person above 60 years	No (ref)								
	Yes	0.51	0.14	0.21	1.24				
Education	Lower secondary/upper secondary (ref)								
	College								
	University					0.20	0.054	0.04	1.02
	Postgraduation								
Profession	Medical doctor (ref)								
	Nurse and midwife								
	Medical technician	0.39	0.028*	0.17	0.90	68.89	0.000 ***	7.33	646.98
Medical specialty	Internal medicine (ref)								
	Surgery	3.22	0.05	1.00	10.35				
	Infectious disease	0.51	0.162	0.20	1.30	0.13	0.044 *	0.01	0.94
	Resuscitation and emergency medicine	37.61	0.066	0.79	1787.41	0.04	0.046 *	0.002	0.94
	Anesthesiology					0.15	0.179	0.01	2.32
	Others					0.07	0.01 *	0.009	0.53
Alcohol	No (ref)								
	Yes					6.83	0.014 *	1.48	31.58
Smoking	No (ref)								
	Yes					0.16	0.101	0.02	1.41
Comorbidities	No (ref)								
	Yes					0.36	0.168	0.08	1.53
Using pain relief medications	No (ref)								
	Yes	9.83	0.111	0.59	163.55	25.50	0.047 *	1.04	620.52
**Psychological trauma-related variables in the past one week**									
Having experienced traumatic stress following a family event	No (ref)								
	Yes					130.32	0.001 **	7.06	2404.04
Having experienced traumatic stress following a work event	No (ref)								
	Yes	46.24	0.000 ***	9.12	234.28	181.55	0.001 **	8.80	3745.22
Having experienced traumatic stress following an academic event	No (ref)								
	Yes	0.06	0.053	0.004	1.03	0.01	0.113	0.00004	2.90
Having experienced traumatic stress following a social event	No (ref)								
	Yes								
Having experienced traumatic stress following a disease event	No (ref)								
	Yes	26.04	0.058	0.89	754.53				
Having experienced traumatic stress following an economic event	No (ref)								
	Yes					4.79	0.164	0.52	43.53
**COVID-19 control and prevention-related variables**									
Feeling with COVID-19 infection risk	No risk (ref)								
	Low risk					5.05	0.071	0.87	29.28
	Average risk								
	High risk					3.89	0.128	0.67	22.45
	Very high risk					29.64	0.011 *	2.20	398.16
	Infected	0.02	0.04 *	0.0005	0.83				
Knowledge preparation before participating in COVID-19	No (ref)								
	Yes	0.19	0.053	0.03	1.02	0.008	0.014 *	0.0002	0.37
Severity of COVID-19 patient	Normal level (ref)								
	Mild level								
	Moderate level					6.46	0.038 *	1.10	37.78
	Severe level	2.16	0.09	0.88	5.27	18.96	0.004 **	2.52	142.41
Full equipment in current workplace conditions	No (ref)								
	Yes					26.68	0.061	0.86	825.40
Duration participating in COVID-19 control (months)	<1 month (ref)								
	1–3 month(s)					0.16	0.089	0.02	1.30
	>3 months	0.52	0.147	0.22	1.25	0.22	0.14	0.03	1.62
Affected a lot by the community	No (ref)								
	Yes					6.33	0.003 **	1.89	21.19
Affected by workplace conditions	No (ref)								
	Yes	5.36	0.000 ***	2.41	11.92				
Pseudo R2		0.3520				0.5654			

OR: odd ratio; *, **, ***: significant at 0.05, 0.01 and 0.001.

**Table 6:** Analysis of factors associated with the symptoms of anxiety: univariable and multivariable logistic regression.

		Clinically Significant Anxiety		Univariate				Multivariate			
		No (*N* = 155)	Yes (*N* = 53)	OR	*p*-Value	Confidence Interval 95%		OR	*p*-Value	Confidence Interval 95%	
						Lower	Upper			Lower	Upper
**Profession-Related and Socio-Demographic Variables**											
Age (Mean; SD)		33.74 (6.87)	31.62 (6.27)	0.95	0.051	0.90	1.00	0.95	0.56	0.83	1.10
Gender	Male (ref)	55	24								
	Female	100	29	0.66	0.206	0.35	1.25	0.68	0.668	0.12	3.85
Marital status	Married (ref)	122	34								
	Single	25	17	2.44	0.016 *	1.18	5.03	7.28	0.046 *	1.03	51.24
	Widowed/Divorced	8	2	0.89	0.894	0.18	4.42	18.90	0.162	0.30	1159.24
Number of people living with (people)	1–3 people (ref)	28	15								
	4–5 people	90	24	0.49	0.077	0.23	1.07	0.22	0.119	0.03	1.47
	>5 people	37	14	0.70	0.438	0.29	1.70	2.26	0.494	0.21	23.67
Family household with own children under 18 years	No (ref)	44	24								
	Yes	111	29	0.47	0.025*	0.25	0.91	0.62	0.625	0.09	4.19
Family household with own older person above 60 years	No (ref)	112	39								
	Yes	43	14	0.93	0.852	0.46	1.89	0.50	0.438	0.08	2.84
Education	Lower secondary/upper secondary (ref)	8	2								
	College	65	30	1.84	0.455	0.36	9.22	0.81	0.918	0.01	43.69
	University	50	14	1.12	0.893	0.21	5.88	0.11	0.328	0.001	8.51
	Postgraduation	32	7	0.87	0.881	0.15	5.04	0.22	0.5	0.003	16.92
Profession	Medical doctor (ref)	43	14								
	Nurse and midwife	89	31	1.06	0.856	0.51	2.21	0.67	0.792	0.03	12.96
	Medical technician	23	8	1.06	0.897	0.39	2.91	42.46	0.058	0.87	2054.21
Medical specialty	Internal medicine (ref)	13	3								
	Surgery	14	10	3.09	0.138	0.69	13.80	1.34	0.865	0.04	39.44
	Infectious disease	47	12	1.106	0.888	0.27	4.51	0.21	0.391	0.006	7.05
	Resuscitation and emergency medicine	13	6	2.00	0.391	0.40	9.75	0.03	0.164	0.0004	3.77
	Anesthesiology	7	4	2.47	0.312	0.42	14.34	0.20	0.476	0.002	15.44
	Others	61	18	1.27	0.723	0.32	4.98	0.07	0.155	0.002	2.67
Alcohol	No (ref)	81	23								
	Yes	74	30	1.42	0.266	0.76	2.67	6.61	0.058	0.93	46.79
Smoking	No (ref)	132	47								
	Yes	23	6	0.73	0.525	0.28	1.90	0.14	0.102	0.01	1.46
Comorbidities	No (ref)	82	26								
	Yes	73	27	1.16	0.629	0.62	2.17	0.29	0.214	0.04	2.03
Using pain relief medications	No (ref)	154	48								
	Yes	1	5	16.04	0.012 *	1.82	140.68	50.00	0.061	0.83	2993.35
**Psychological trauma- related variables in the past one week**											
Having experienced traumatic stress following a family event	No (ref)	149	37								
	Yes	6	16	10.73	0.000 ***	3.93	29.33	153.97	0.003	5.43	4362.13
Having experienced traumatic stress following a work event	No (ref)	148	29								
	Yes	7	24	17.49	0.000 ***	6.89	44.40	265.42	0.002 **	8.39	8389.72
Having experienced traumatic stress following an academic event	No (ref)	152	46								
	Yes	3	7	7.71	0.004 **	1.91	31.02	0.01	0.14	0.00002	4.44
Having experienced traumatic stress following a social event	No (ref)	149	44								
	Yes	6	9	5.07	0.003 **	1.71	15.05	0.003	0.014 *	0.00004	0.32
Having experienced traumatic stress following a disease event	No (ref)	154	47								
	Yes	1	6	19.65	0.006 **	2.30	167.43	N/A			
Having experienced traumatic stress following an economic event	No (ref)	141	30								
	Yes	14	23	7.72	0.000 ***	3.56	16.71	5.28	0.174	0.47	58.26
**COVID-19 control and prevention-related variables**											
Severity of COVID-19 patient	Normal level (ref)	25	7								
	Mild level	38	3	0.28	0.086	0.06	1.19	0.40	0.57	0.01	9.11
	Moderate level	56	18	1.14	0.785	0.42	3.09	3.57	0.37	0.22	58.05
	Severe level	36	25	2.48	0.070	0.92	6.61	15.56	0.052	0.97	248.97
Duration participating in COVID-19 control (months)	<1 month (ref)	21	10								
	1–3 month(s)	52	10	0.40	0.079	0.14	1.11	0.09	0.062	0.008	1.12
	>3 months	82	33	0.84	0.700	0.35	1.98	0.11	0.088	0.009	1.38
Knowledge preparation before participating in COVID-19	No (ref)	5	7								
	Yes	150	46	0.21	0.013 *	0.06	0.72	0.02	0.101	0.0002	2.11
Full equipment in current workplace conditions	No (ref)	10	3								
	Yes	145	50	1.14	0.837	0.30	4.34	20.60	0.106	0.52	803.78
Affected by workplace conditions	No (ref)	106	22								
	Yes	49	31	3.04	0.001 **	1.60	5.79	1.60	0.521	0.37	6.75
Affected a lot by the community	No (ref)	111	18								
	Yes	44	35	4.90	0.000 ***	2.51	9.55	6.13	0.016 *	1.40	26.84
Feeling with COVID-19 infection risk	No risk (ref)	17	5								
	Low risk	45	10	0.75	0.650	0.22	2.53	5.59	0.286	0.23	132.36
	Average risk	41	13	1.07	0.900	0.33	3.49	1.46	0.814	0.06	35.46
	High risk	36	13	1.22	0.734	0.37	4.00	5.41	0.299	0.22	131.15
	Very high risk	14	12	2.91	0.096	0.82	10.27	33.29	0.078	0.67	1647.01
	Infected	2	0	N/A				N/A			
Having a relative/friend/ colleague with positive COVID-19	No (ref)	76	22								
	Yes	79	31	1.35	0.344	0.72	2.54	0.87	0.875	0.17	4.51
Pseudo R2								0.5838			

OR: odd ratio; *, **, ***: significant at 0.05, 0.01 and 0.001.

## Data Availability

The data used to support the findings of this study are available from the author Hoang-Long Vo (H.-L.V.) upon request (Email: vohoanglonghmu@gmail.com).
